# Designing Virtual Reality Game-Based Learning for Computational Thinking Competency Development: Evidence from Vocational Education

**DOI:** 10.12688/f1000research.183507.1

**Published:** 2026-10-05

**Authors:** Dias Aziz Pramudita, Ratna Wardani, Mochamad Bruri Triyono, Sukirman Sukirman, Roudlotus Sholikhah, Adira Bintang Permana, Anis Khoerun Nisa, Thomas Köhler

**Affiliations:** 1 Technology and Vocational Education, Universitas Negeri Yogyakarta Program Pascasarjana, Yogyakarta, Special Region of Yogyakarta, 55281, Indonesia; 2 Informatics Engineering Education, Universitas Muhammadiyah Surakarta, Surakarta, Central Java, 57169, Indonesia; 3 Educational Technology, TU Dresden Faculty of Educational Studies, Dresden, Saxony, 01062, Germany; 4 Fashion Education Department, Faculty of Engineering, Universitas Negeri Semarang, Semarang, Indonesia; 5 Muhammadiyah Al-Kautsar Junior High School, Indonesia, Central Java, Indonesia; 6 Department of Software and Game Development, State Vocational High School 5 Surakarta, Surakarta, Indonesia

**Keywords:** virtual reality, game-based learning, computational thinking, competency development, vocational education, immersive learning

## Abstract

Computational thinking has become an essential skill in vocational education; however, many instructional approaches struggle to deliver authentic, engaging, and practice-oriented learning experiences. Virtual reality (VR) game-based learning presents a promising solution to address these challenges by blending immersive environments with problem-solving activities that support the development of competencies. This study explores the design and effectiveness of a VR game-based learning environment to enhance computational thinking skills in vocational education. A quasi-experimental research design was implemented, involving vocational high school students who participated in a VR-based game learning intervention integrated into their regular instructional framework. To assess computational thinking competencies, pre- and post-tests were conducted, aligned with the core components of computational thinking, and supplemented by performance-based learning tasks. The findings reveal that students experienced significant improvements in their computational thinking skills following the intervention, indicating that the VR game-based learning environment effectively supported competency development. Furthermore, the results emphasize the importance of incorporating authentic vocational scenarios and game mechanics to foster active engagement and practical problem-solving. This study contributes valuable empirical evidence to the growing body of research on game-based learning, demonstrating how thoughtfully designed virtual reality learning environments can enhance the development of computational thinking competencies in vocational education contexts.

## Introduction

Computational thinking (CT) is now widely framed as a core 21st-century competence that underpins problem solving, programming, and adaptability in digitalized workplaces, and is therefore increasingly embedded in school and vocational curricula. ^1^ ^–^ ^3^ In vocational education and training (VET), where the mission is to develop practice-oriented, occupation-specific competencies, CT is viewed as a key enabler for operating cyber-physical systems, automation, and data-driven processes across sectors such as manufacturing, automotive, and healthcare. ^4^ ^–^ ^7^ However, many existing CT teaching approaches still rely on abstract tasks and de-contextualized programming exercises that fail to reflect the authentic, tool-rich problem contexts of vocational practice, limiting learners’ motivation and transfer of skills. ^8^ ^,^ ^9^

Immersive virtual reality (VR) has emerged as a promising technology to close this gap by enabling realistic simulations of work processes and interactive, first-person engagement with digital tools and environments. ^5^ ^,^ ^9^ ^–^ ^11^ Systematic reviews and bibliometric analyses show a steep rise in VR adoption in vocational contexts over the last decade, with research highlighting VR’s potential to provide safe, repeatable practice, reduce material costs, and support complex procedural learning in domains such as welding, electrical systems, and healthcare. ^5^ ^,^ ^12^ Within CT education, VR has been combined with game-based learning (GBL) in “game-based in VR” (GBiVR) designs that exploit immersion, presence, and interactivity to foster CT skills through puzzles and problem scenarios. ^13^ ^–^ ^15^

Recent empirical studies report that VR game-based environments can improve CT or closely related skills such as algorithmic thinking, recursive reasoning, and systematic problem solving, compared with traditional or non-immersive conditions. For example, the iThinkSmart mini-game suite in immersive VR led to higher CT competence, increased motivation, and more positive attitudes toward CT learning than a 2D technology-enhanced approach in higher education computer science students. ^15^ Structural-equation modeling with the same environment showed that specific game elements—particularly goal clarity and feedback—and VR features such as immersion and interaction significantly predicted students’ reflective thinking and comprehension, emphasizing the importance of careful pedagogical and interaction design rather than technology alone. ^3^ In vocational and low-resource settings, low-budget VR game environments for CT have been found usable and well accepted by vocational high-school students, with perceived ease of use and usefulness positively linked to willingness to reuse VR-based CT tools. ^16^ A more recent CT Saber VR game achieved significant pre- to post-test gains in CT skills among university computer science students, reinforcing the view that VR mini-games can effectively scaffold CT practice. ^1^

At the same time, broader reviews of VR and mixed-reality training in education and VET underscore that learning benefits are not automatic. Meta-analyses of immersive VR and mixed reality report medium to large overall effects on cognitive, behavioral, and affective outcomes, but also highlight strong heterogeneity and a lack of comparative instructional research. ^17^ ^,^ ^18^ Systematic reviews on VR and gamification in education conclude that gamified VR environments tend to improve engagement, motivation, and achievement compared with traditional instruction, yet they also identify challenges such as motion sickness, cognitive overload, accessibility barriers, and the need for robust instructional design aligned with learning objectives. ^10^ ^,^ ^19^ ^–^ ^21^ In VET specifically, emerging reviews stress that much VR work remains technology-driven, with relatively little focus on pedagogical models, authentic workplace integration, or longitudinal competency development. ^5^ ^,^ ^22^ ^,^ ^23^

Unlike general or K-12 education, where computational thinking (CT) is often taught using abstract logic puzzles or generic block-based coding, VET requires a domain-specific and practice-oriented framework. ^24^ In VET environments, CT serves as an applied enabler for operating, debugging, and building technical systems. ^25^ To reflect the peculiar characteristics of VET, the instructional design must satisfy three key criteria: (a) authentic workplace contextualization ^26^; (b) tool-rich interaction with simulated technical apparatus; and (c) a competency-oriented focus linking abstract logic to vocational practice.

For vocational education, this raises two open questions. First, how can VR game-based environments be designed to reflect authentic vocational tasks and processes, rather than generic logic puzzles, so that CT is developed in close connection with domain-specific practice ^4^ ^,^ ^5^ ^,^ ^27^? Second, to what extent do such embedded, practice-oriented VR game scenarios support measurable gains in CT competencies for vocational learners, beyond usability, motivation, or self-report outcomes ^1^ ^,^ ^3^? Existing CT-oriented VR games such as iThinkSmart and CT Saber have been evaluated primarily with university computer science cohorts, ^13^ ^,^ ^15^ while systematic reviews of AR/VR for CT show that research is dominated by higher education computer science and mathematics, with comparatively few studies targeting vocational tracks or non-academic CT applications. ^28^ In the wider VET literature, VR is often applied to technical or safety training without explicit CT constructs or assessments, leaving unclear how far learners’ CT competencies develop through such experiences. ^5^ ^,^ ^27^

There is also ongoing debate about the relative added value of immersive VR compared with non-immersive game-based platforms for higher-order thinking. Comparative work in digital game-based learning suggests that VR can yield stronger gains in certain critical-or computational-thinking dimensions, such as interpretation or spatial reasoning, but that platform effects may interact with task design and learner characteristics. ^18^ ^,^ ^29^ ^,^ ^30^ Mixed-reality meta-analyses note that, when controlling for instructional content, differences among MR modalities can diminish, implying that factors such as scaffolding, feedback, and practice opportunities may be more decisive than immersion alone. ^17^ In CT-focused VR game research, some authors warn that poorly designed VR tasks may introduce extraneous cognitive load or distraction, potentially offsetting benefits of presence and engagement. ^31^

Against this backdrop, vocational education faces a dual imperative: to cultivate CT as a transversal competence for Industry 4.0 workplaces, and to do so through learning experiences that are authentic, engaging, and closely aligned with real work processes. ^4^ ^,^ ^6^ ^,^ ^22^ Recent VET-specific reviews show that game-based and gamified approaches can enhance motivation and academic performance in vocational learners, particularly in engineering and healthcare programs, but they also call for more research on how game mechanics and digital simulations can be systematically linked to competency frameworks and assessment. ^4^ Reviews of immersive VR in vocational schools similarly identify strong potential for improving learning outcomes and motivation, while emphasizing unresolved issues such as motion sickness, cognitive load, and the scarcity of studies that embed VR in full courses or teaching-factory models. ^10^ ^,^ ^27^

The present study responds to these gaps by designing and empirically evaluating a VR game-based learning environment explicitly targeted at developing CT competencies in vocational high-school students. Building on conceptual frameworks for CT-oriented game-based VR ^13^ ^,^ ^28^ on design science approaches used in prior CT-VR systems ^1^ ^,^ ^15^), the environment integrates authentic vocational scenarios with game mechanics aligned to core CT dimensions such as decomposition, abstraction, algorithmic thinking, and debugging. Within a quasi-experimental design embedded in regular instruction, CT competencies are assessed through pre−/post-tests and performance-based tasks to capture both conceptual understanding and applied problem solving. By focusing on vocational high-school learners and situating CT challenges in realistic work-like contexts, this study aims to contribute (a) empirical evidence on the effectiveness of VR game-based learning for CT development in VET, and (b) design insights on how authentic vocational scenarios and specific game/VR features can be orchestrated to support active engagement and practice-oriented CT competence development. ^5^ ^,^ ^27^ ^,^ ^32^

## Methods

### Research design

This study employed a quasi-experimental pretest–posttest research design to assess the effectiveness of a virtual reality (VR) game-based learning environment in enhancing computational thinking (CT) skills among vocational high school students. The focus on CT is crucial in today’s digital age, as it equips students with the problem-solving and logical reasoning abilities necessary for success in various fields. The intervention involved a carefully designed VR game seamlessly integrated into the school curriculum’s regular instructional framework. This approach allowed students to engage in learning activities that were not only immersive and interactive but also took place in authentic classroom settings. By maintaining ecological validity, the study ensured that the educational experience reflected real-world conditions students would encounter, thereby promoting greater engagement and retention of skills. The pre- and post-tests measured students’ CT competencies before and after the intervention, providing valuable insights into the impact of the VR-based learning environment on their development. Through this comprehensive approach, the study aimed to advance understanding of innovative teaching methods in vocational education.

Participants took a CT pre-test before the intervention to determine their baseline competency levels. They then participated in a series of VR game-based learning sessions designed around real-world vocational problem scenarios and aligned with key computational thinking (CT) dimensions, such as decomposition, abstraction, algorithmic thinking, and debugging. After completing the intervention, participants underwent a post-test and performance-based learning tasks to measure changes in their CT competencies and applied problem-solving performance.

This quasi-experimental approach was selected because random assignment of students to experimental and control groups was not feasible within the existing school timetable and curricular structure. Instead, intact classes were used, reflecting common constraints in vocational education research while enabling the examination of learning outcomes in a realistic educational setting. The design allowed for the analysis of within-group changes in CT competencies attributable to the VR game-based learning experience.

### Participants & context

The participants in this study were vocational high-school students enrolled in the Software Engineering and Game Development Department (PPLG) at both public and private vocational schools located in the Solo Raya region, Indonesia. The study targeted approximately 40 vocational high-school students from intact PPLG classes. The PPLG program focuses on software development, programming, and digital application design, making it a relevant vocational context for the development of computational thinking competencies.

Participants were drawn from intact classes as part of their regular instructional activities. This sampling approach was adopted to accommodate institutional and scheduling constraints commonly encountered in vocational education settings. All participating students had prior exposure to basic computing and introductory programming concepts through their formal curriculum but had no prior experience with immersive VR-based game learning before the study.

The VR game-based learning intervention was implemented within a scheduled instructional period and integrated into the existing course structure to ensure ecological validity. Learning activities were conducted in a school environment under teacher supervision, with students engaging individually in the VR learning tasks. The instructional context emphasized applied problem solving and practice-oriented learning, reflecting the competency-based nature of vocational education and training.

### VR Game-based learning environment (intervention)

**Design framework & learning objectives**

The intervention consisted of a virtual reality (VR) game-based learning environment explicitly designed to support the development of computational thinking (CT) competencies in a vocational education context. The design of the VR learning environment was informed by established computational thinking frameworks and prior research on game-based learning in immersive VR, which emphasize the alignment of learning objectives, game mechanics, and interaction design to avoid technology-driven rather than pedagogy-driven implementations.

The learning objectives targeted core CT dimensions commonly identified in the literature, namely problem decomposition, abstraction, algorithmic thinking, and debugging. These competencies were operationalized through authentic vocational problem scenarios relevant to the PPLG program, ensuring that CT development was situated within realistic, practice-oriented tasks rather than abstract or decontextualized programming exercises. To address the distinct requirements of vocational education compared to non-vocational CT interventions, ^24^ the VR game design incorporated specific VET-aligned criteria:

a. **Contextualized Task Scenarios**: Rather than presenting generic logic puzzles, in-game challenges were explicitly contextualized into authentic tasks relevant to the PPLG curriculum. ^26^
b. **First-Person Tool Interaction**: Learners interacted directly with virtual objects and system interfaces, simulating the hands-on, tool-rich environment characteristic of vocational training. ^25^
c. **Performance-Based Applied Feedback**: Immediate visual and quantitative feedback was provided within the simulated system, reinforcing procedural accuracy and real-time troubleshooting skills essential for Industry 4.0 technical occupations.

In addition, the design of the VR game-based learning environment was informed by the Bebras Computational Thinking Challenge, which provides well-established task types targeting core CT dimensions. The in-game problem-solving missions were developed by adapting Bebras-style tasks and embedding them within immersive, vocationally relevant scenarios. While the underlying logical structures and cognitive demands of the Bebras tasks were preserved, the tasks were contextualized to reflect authentic work-like situations relevant to the PPLG program.

**Game mechanics & learning activities**

Learning activities were structured as problem-solving missions adapted from Bebras Computational Thinking Challenge tasks, in which students interacted with virtual objects, tools, or systems to complete tasks aligned with specific computational thinking dimensions. These mechanics included clear goals and task instructions, progressive challenge levels, immediate feedback, and task completion indicators. Each game level presented learners with a vocationally grounded problem scenario that required systematic reasoning and decision making.

Learning activities were structured as problem-solving missions, in which students interacted with virtual objects, tools, or systems to complete tasks aligned with specific computational thinking dimensions. For example, learners were required to break down complex tasks into manageable steps (decomposition), identify relevant information while ignoring extraneous elements (abstraction), arrange actions in logical sequences (algorithmic thinking), and identify recurring structures or similarities across tasks to inform solution strategies (pattern recognition).

The game progression was designed to gradually increase task complexity, allowing learners to build confidence and competence while minimizing cognitive overload. Feedback was provided through in-game visual cues and task outcomes, enabling learners to reflect on their actions and adjust their strategies accordingly.

**VR Technology & hardware**

The VR game-based learning environment was implemented using an immersive virtual reality system based on the Meta Quest 2 headset, a stand-alone head-mounted display suitable for classroom-based deployment in vocational education settings. The Meta Quest 2 was selected due to its portability, ease of use, and ability to operate without an external computer, making it feasible for use in both public and private vocational schools.

Students interacted with the VR environment individually using the Meta Quest 2 head-mounted display and handheld motion controllers, which supported first-person interaction, object manipulation, selection, and navigation within the virtual space. The interaction design emphasized task-oriented actions and tool use relevant to vocational problem solving, rather than free exploration, to minimize extraneous cognitive load and reduce the risk of motion sickness.

### Procedure

The study followed a structured quasi-experimental pre-test–intervention–post-test procedure. Prior to the intervention, participants completed a computational thinking pre-test to establish baseline competency levels across the targeted CT dimensions. The pre-test was administered during a regular class session under teacher supervision.

Before the first VR learning session, students participated in a brief familiarization and training session to introduce the VR equipment and interaction mechanics. This session included instructions on the safe use of the Meta Quest 2 headset, basic navigation and object manipulation using handheld controllers, and an overview of the game-based learning tasks. The purpose of this orientation was to reduce the influence of technical novelty on learning outcomes.

The VR game-based learning intervention was then implemented during scheduled instructional sessions. Students engaged with the VR environment individually, completing a series of problem-solving missions embedded in authentic vocational scenarios. Each VR session lasted approximately 30–45 minutes, and the intervention was conducted over multiple sessions integrated into the regular course schedule. During the sessions, the instructor acted primarily as a facilitator, providing technical assistance and classroom management support when needed, while avoiding direct guidance related to task solutions or strategies.

Following completion of the VR learning sessions, participants completed a post-test using the same computational thinking instrument administered at pre-test. In addition, students undertook performance-based learning tasks designed to assess the application of computational thinking competencies in vocationally relevant problem contexts. The post-test and performance tasks were administered under the same conditions as the pre-test to ensure consistency and support internal validity.

### Instruments & measures

**Computational thinking test**

Computational thinking (CT) competency was measured using a test instrument adapted from the Bebras International Challenge on Informatics and Computational Thinking. The Bebras Challenge is an internationally recognized initiative that provides validated task formats designed to assess core computational thinking dimensions in an age-appropriate and context-neutral manner.

The instrument used in this study consisted of 10 scenario-based multiple-choice items, each presenting a short problem-solving situation requiring logical reasoning rather than programming syntax knowledge. The tasks were adapted and contextualized to align with the vocational Software Engineering and Game Development (PPLG) curriculum while preserving the underlying logical structure and cognitive demand of the original Bebras task types. The test assessed four core CT dimensions as mentioned in Table 1.

**Table 1.** CT dimensions.

No	Dimension	Description
1	Decomposition	Breaking down complex problems into manageable components
2	Abstraction	Identifying relevant information while ignoring irrelevant details
3	Algorithmic Thinking	Constructing and evaluating logical sequences of steps
4	Pattern Recognition	Identifying recurring structures, similarities, or regularities across problem situations

Each item had four answer options with one correct response. Correct answers were scored as 1 and incorrect answers as 0, yielding a total possible score range of 0–10.

Content validity was supported through alignment with established Bebras task typologies and review by two subject-matter experts in computer science education. A pilot administration was conducted with a small group of students from a comparable vocational cohort to evaluate item clarity and difficulty level. Internal consistency reliability was evaluated using Cronbach’s alpha coefficient during the main study data analysis phase.

The same instrument was administered as both pre-test and post-test to measure changes in CT competency over the course of the VR intervention.

**Performance-based vocational tasks**

To complement the paper-based CT test and capture applied computational thinking in authentic contexts, students completed performance-based vocational tasks following the intervention.

These tasks required learners to solve open-ended, vocationally grounded problems within structured scenarios reflecting software and game development contexts. Tasks were designed to assess the application of CT dimensions in practical situations, including:

1. Structuring a solution plan for a simulated software feature
2. Identifying logical errors in a simplified workflow model
3. Organizing system components into coherent functional sequences

Performance tasks were evaluated using an analytic scoring rubric developed specifically for this study. The rubric assessed four criteria aligned with CT dimensions which are Problem Decomposition, Logical Sequencing and Algorithm Design, Abstraction and Pattern Recognition. As can be seen in Table 2, each criterion was scored on a 4-point scale (0–3) ranging from “limited evidence of competency” to “highly proficient and systematic application.” Total rubric scores ranged from 0 to 12.

**Table 2.** Rubric descriptors for performance task.

No	Score	Description
1	0	No evidence of competency
2	1	Limited or fragmented application
3	2	Adequate but partially systematic application
4	3	Highly systematic and accurate application

To enhance scoring reliability, rubric descriptors were defined explicitly. If multiple raters were involved, inter-rater reliability was assessed using agreement coefficients prior to final score consolidation. The inclusion of performance-based tasks allowed triangulation of findings and strengthened the ecological validity of the measurement of CT competency beyond selected-response testing.

### Data analysis

Descriptive statistics (mean, standard deviation, minimum, and maximum scores) were calculated for pre-test and post-test results. Assumptions of normality were assessed using distribution diagnostics to determine the appropriateness of parametric statistical testing. To evaluate within-group differences between pre-test and post-test CT scores, a paired-samples t-test was conducted if normality assumptions were satisfied. In cases where normality was violated, a non-parametric alternative (Wilcoxon signed-rank test) was applied. Statistical significance was evaluated at an alpha level of p < .05.

Effect sizes were calculated using Cohen’s d to estimate the magnitude of the intervention’s impact on CT competency. Effect size interpretation followed conventional benchmarks (small, medium, large). For performance-based task scores, descriptive statistics were computed to assess overall competency levels. Where appropriate, correlational analysis was conducted to examine the relationship between post-test CT scores and performance-based task outcomes, providing additional evidence of construct alignment. All analyses were conducted using established statistical software to ensure accuracy and reproducibility.

### Materials, data & code availability

The VR game-based learning environment, including adapted task structures derived from the Bebras International Challenge on Informatics and Computational Thinking framework, was developed for research purposes. Due to licensing considerations related to adapted Bebras task content, the full game build is not publicly distributed in its original form.

De-identified datasets generated and analyzed during the current study will be made available in a publicly accessible institutional or open research repository upon acceptance of the manuscript. Access details and persistent identifiers (e.g., DOI) will be provided prior to publication.

The computational thinking test instrument (adapted items), scoring rubric for performance-based tasks, and analysis scripts will be made available as supplementary materials to facilitate replication and further research. Any restrictions related to third-party intellectual property are disclosed at submission in accordance with journal policy.

**Use of generative AI**

Generative artificial intelligence (GenAI) tools were used to assist in language refinement and structural organization of the manuscript. All study design decisions, intervention development, data collection procedures, statistical analyses, and interpretation of findings were conducted by the authors. No generative AI tools were used to fabricate data, generate empirical results, or automate data analysis.

## Results

### Implementation and design validation of the VR game-based learning environment

The VR game-based learning environment was implemented within intact Software Engineering and Game Development (PPLG) classes in vocational high schools in the Solo Raya region, Indonesia. The intervention was conducted during scheduled instructional sessions using the Meta Quest 2 head-mounted display, with students engaging individually in structured problem-solving missions.

As shown in Figure 1, the VR application consists of progressive missions targeting four computational thinking (CT) dimensions: decomposition, abstraction, algorithmic thinking, and pattern recognition. Task structures were adapted from the Bebras International Challenge on Informatics and Computational Thinking and contextualized within vocationally relevant software development and game design scenarios aligned with the PPLG curriculum.

**Figure 1.**
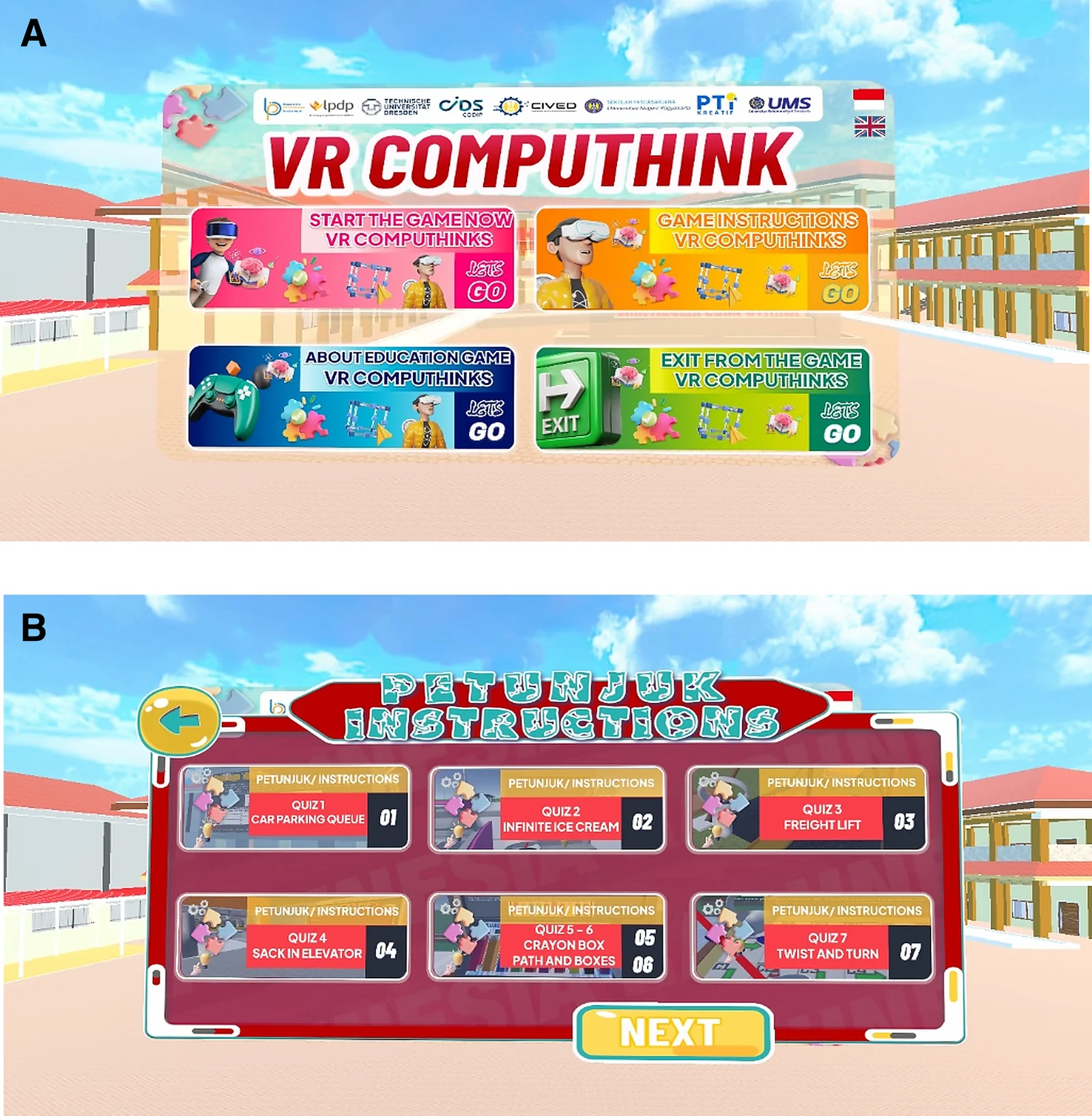
The interface and problem-solving mission panels of the VR CompuThink educational game. (A) Homepage panel of the VR game, displaying the primary navigation options including game start, instructions, educational information, and language options. (B) Problem-solving task missions interface, presenting the structured levels and computational thinking challenges (e.g., car parking queue, freight lift, and elevator-based optimization tasks) designed for student engagement and assessment.

The missions were sequenced to increase in cognitive complexity, beginning with guided decomposition and sequencing tasks and progressing toward independent pattern identification and multi-step reasoning challenges as illustrates in Figure 2. This structure was intended to scaffold higher-order thinking while maintaining alignment with vocational problem-solving practices.

**Figure 2.**
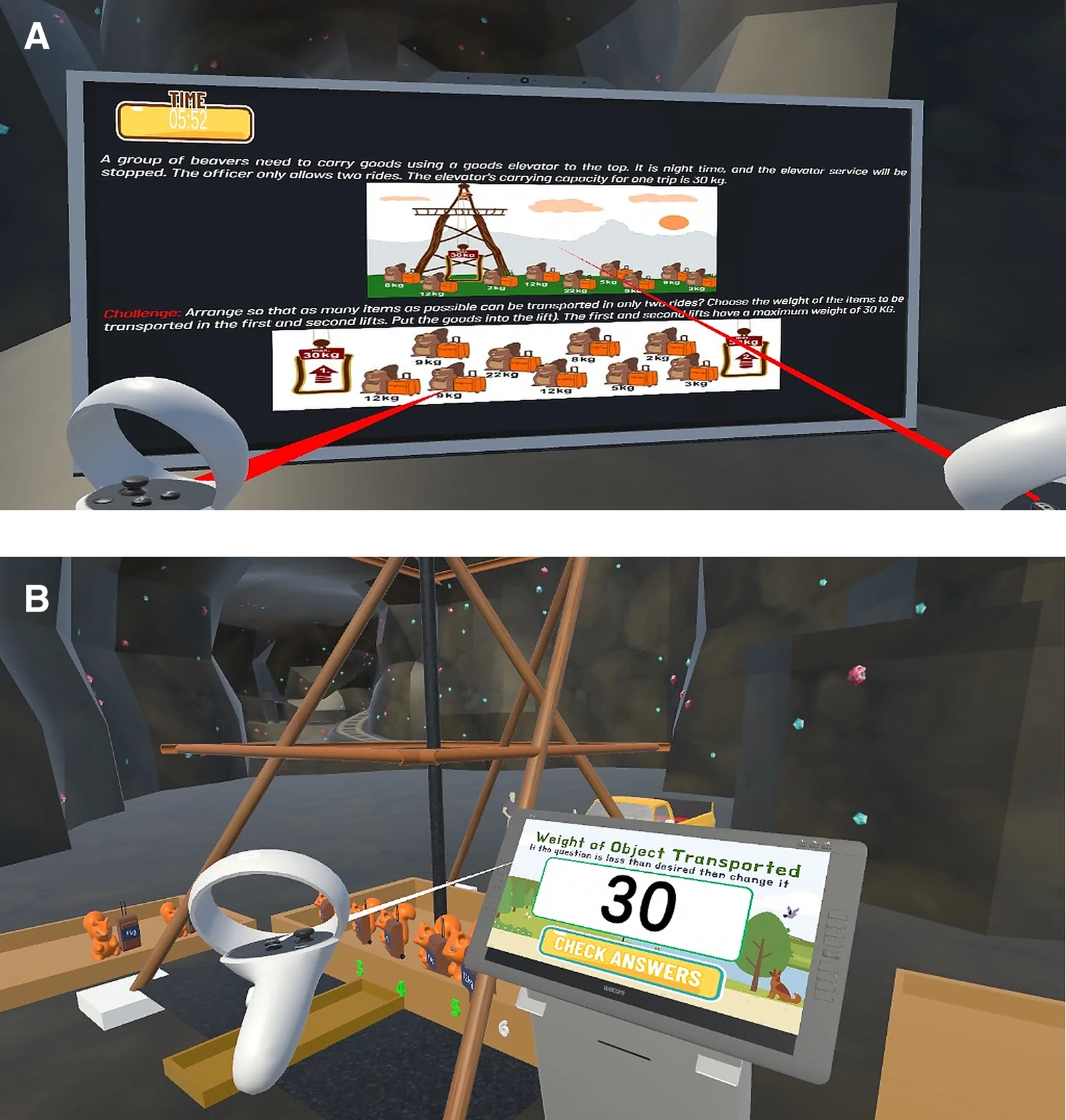
In-game problem-solving mechanics and real-time interaction panels in the VR CompuThink environment. (A) Freight lift and elevator access challenge panel, illustrating the computational thinking problem scenario where users are tasked with optimizing item transportation under weight and capacity constraints within an allotted time. (B) First-person interactive VR view using Meta Quest controllers, showing the real-time display monitor that reflects the accumulated weight of the transported objects along with interactive submission controls.

As can be seen from Figure 3, students completed the assigned missions within the allocated 30–45-minute sessions. Following the initial orientation, participants were able to navigate the VR environment effectively despite having no prior experience with immersive VR learning. Observational monitoring indicated consistent task-focused engagement throughout implementation.

**Figure 3.**
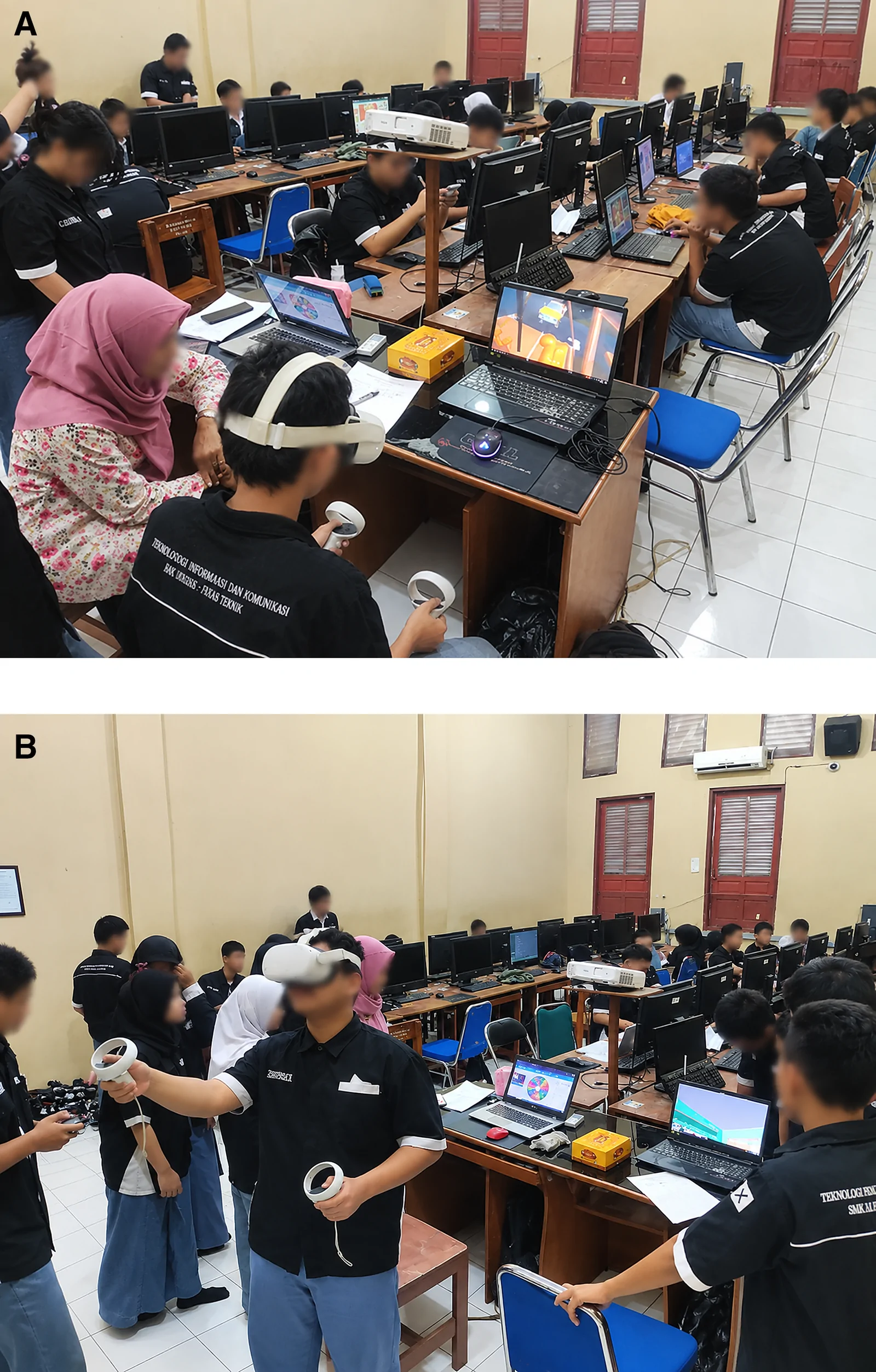
Classroom implementation and student interaction during the VR CompuThink learning session. (A) A student immersed in solving the computational thinking challenge using the VR headset and hand controllers under direct observation and guidance by the teacher. (B) Students actively engaged in the VR learning activity, demonstrating the rotation workflow where individual participants take turns operating the VR equipment while peers monitor the mirrored gameplay and problem-solving progress on computer screens. (Note: All recognizable faces have been blurred for ethical compliance and participant de-identification in accordance with editorial policies).

Overall, the intervention demonstrated coherence between pedagogical objectives, vocational relevance, and immersive interaction design, providing an appropriate basis for examining its impact on computational thinking competencies.

### Preliminary statistical analysis

A total of N = 36 vocational high-school students from PPLG classes completed both the pre-test and post-test assessments and were included in the final analysis. As can be seen in Table 3, descriptive statistics indicated that the mean pre-test computational thinking (CT) score was M = 11.42 (SD = 2.31) out of 20 points. Following the VR intervention, the mean post-test score increased to M = 14.87 (SD = 2.48). Internal consistency reliability of the adapted CT instrument was assessed separately for pre-test and post-test administrations. The Cronbach’s alpha coefficient was α = .79 for the pre-test and α = .83 for the post-test, indicating acceptable to good reliability for research purposes. Prior to inferential testing, the assumption of normality was evaluated using the Shapiro–Wilk test on the difference scores (post-test minus pre-test). Results indicated no significant deviation from normality (W = .97, p = .38), supporting the use of parametric statistical procedures in subsequent analyses. Overall, the preliminary analyses confirmed that the instrument demonstrated satisfactory reliability and that the data met the assumptions required for examining the impact of the VR intervention on CT competency.

**Table 3.** Descriptive statistics, internal consistency, and normality test results (N = 36).

Measure	Pre-test	Post-test	Difference (Post-Pre)
Mean	11.42	14.87	3.45
Standard Deviation (SD)	2.31	2.48	1.95
Cronbach’s α	0.79	0.83	-
Shapiro-Wilk (W)	-	-	0.97
p-value (Normality)	-	-	0.38

### Effects of the VR intervention on overall computational thinking competency

Given the normal distribution of difference scores, a paired-samples t-test was conducted to examine whether the VR-based learning intervention significantly improved students’ computational thinking (CT) performance. Results from the Table 4 indicated a statistically significant increase in CT scores from pre-test (M = 11.42, SD = 2.31) to post-test (M = 14.87, SD = 2.48), t(35) = 9.12, p < .001.

**Table 4.** Paired-samples t-test results for computational thinking scores (N = 36).

Variable	Pre-test (SD)	Post-test (SD)	Mean Difference	t	df	p	Cohen’s d
CT Score	11.42 (2.31)	14.87 (2.48)	3.45 (1.95)	9.12	35	< 0.001	1.18

The mean gain score was 3.45 points (SD = 1.95), representing a substantial improvement following the intervention. To determine the magnitude of the effect, Cohen’s d for paired samples was calculated. The effect size was large (d = 1.18), indicating a strong practical impact of the VR intervention on students’ CT competency. These findings suggest that immersive VR-based learning significantly enhances computational thinking performance among vocational high-school students.

### Effects of the VR intervention across computational thinking dimensions

To investigate whether the observed improvement in overall computational thinking (CT) performance was reflected across specific CT components, paired-samples t-tests were conducted for Decomposition, Pattern Recognition, Abstraction, and Algorithmic Thinking.

Following Table 5, results indicated statistically significant improvements across all four dimensions following the VR intervention. For Decomposition, mean scores increased from 2.91 (SD = 0.88) at pre-test to 3.74 (SD = 0.92) at post-test, t(35) = 6.84, p < .001, with a large effect size (d = 1.14). For Pattern Recognition, scores improved from 2.85 (SD = 0.91) to 3.68 (SD = 0.89), t(35) = 6.21, p < .001, yielding a large effect (d = 1.03). For Abstraction, the mean increased from 2.73 (SD = 0.95) to 3.55 (SD = 0.93), t(35) = 5.94, p < .001, with a large effect size (d = 0.99). For Algorithmic Thinking, scores rose from 2.93 (SD = 0.87) to 3.90 (SD = 0.85), t(35) = 7.12, p < .001, demonstrating the strongest improvement among the dimensions (d = 1.19).

**Table 5.** Paired-samples t-test results across computational thinking dimensions (N = 36).

Dimension	Pre-test (SD)	Post-test (SD)	Mean difference	t	df	p	Cohen’s d
Decomposition	2.91 (0.88)	3.74 (0.92)	0.83	6.84	35	< 0.001	1.14
Pattern Recognition	2.85 (0.91)	3.68 (0.89)	0.83	6.21	35	< 0.001	1.03
Abstraction	2.73 (0.95)	3.55 (0.93)	0.82	5.94	35	< 0.001	0.99
Algorithmic Thinking	2.93 (0.87)	3.90 (0.85)	0.97	7.12	35	< 0.001	1.19

Overall, the findings suggest that the VR-based intervention produced consistent and substantial gains across all CT components, with particularly pronounced effects in Algorithmic Thinking and Decomposition.

### Performance-based assessment of applied computational thinking

To examine students’ ability to apply computational thinking (CT) in authentic vocational contexts, performance-based tasks were evaluated using the analytic rubric described in the Methods section. Total scores ranged from 0 to 12.

Descriptive analysis indicated that students achieved a mean total performance score of 9.61 (SD = 1.42) as stated in the Table 6, corresponding to 80.1% of the maximum possible score. This suggests that, following the intervention, students were generally able to demonstrate systematic and accurate application of CT processes in open-ended problem-solving scenarios.

**Table 6.** Performance-based assessment results (N = 36).

Dimension	Max Score	Mean	SD	Percentage (%)
Decomposition	3	2.48	0.46	82.7
Algorithm Design	3	2.56	0.41	85.3
Abstraction	3	2.18	0.57	72.7
Pattern Recognition	3	2.39	0.52	79.7
**Total Score**	**12**	**9.61**	**1.42**	**80.1**

At the dimension level, the highest mean score was observed in Logical Sequencing and Algorithm Design (M = 2.56, SD = 0.41), followed by Problem Decomposition (M = 2.48, SD = 0.46) and Pattern Recognition (M = 2.39, SD = 0.52). The comparatively lowest performance was found in Abstraction and Relevance Filtering (M = 2.18, SD = 0.57), although scores remained within the “adequate to systematic application” range.

Overall, the performance-based findings align with the objective test results reported earlier, indicating that improvements in computational thinking were not limited to selected-response measures but extended to applied, vocationally grounded tasks. The convergence of these findings provides additional support for the practical impact of the VR-based intervention on students’ CT competency.

### Synthesis of empirical findings

The present study examined the impact of VR game-based learning on vocational students’ computational thinking (CT) competency using multiple complementary measures. Across objective pre–post testing, dimension-level analyses, and performance-based assessment, findings consistently indicated significant and substantial improvement following the intervention.

At the overall level, students demonstrated a statistically significant increase in CT scores with a large effect size, suggesting that immersive VR-based learning environments can meaningfully enhance computational reasoning skills. Dimension-specific analyses revealed that improvements were distributed across all four CT components—Problem Decomposition, Pattern Recognition, Abstraction, and Algorithmic Thinking—indicating a balanced developmental pattern rather than isolated gains.

Comparative gain analysis further showed that although Algorithmic Thinking exhibited the numerically strongest improvement, differences among dimensions were not statistically significant. This suggests that the intervention supported a holistic development of CT processes.

Importantly, findings from the performance-based vocational tasks converged with the objective test results. Students were able to apply CT principles in authentic, open-ended scenarios situated within software and game development contexts. This triangulation strengthens the validity of the observed learning outcomes and supports the practical relevance of the intervention within vocational education settings. Taken together, the empirical evidence indicates that VR game-based learning can promote both conceptual understanding and applied computational thinking competency among vocational high-school students.

## Discussion

The current study examines the impact of immersive virtual reality (VR) game-based learning on vocational students’ computational thinking (CT) skills. The hypothesis is that this innovative educational approach will significantly enhance their ability to understand and apply computational concepts. The empirical findings robustly support this hypothesis, indicating a marked improvement in students’ overall CT performance after participating in the VR-based intervention and underscoring the effectiveness of integrating technology into vocational training.

From the perspective of prior research, these results are consistent with studies suggesting that immersive and interactive learning environments can promote higher-order cognitive processes, particularly when learners actively engage in problem-solving rather than passively receive instruction. Previous investigations into game-based learning have reported positive effects on analytical reasoning and algorithmic skills, especially when tasks are structured progressively and aligned with authentic contexts. The current findings extend this body of work by demonstrating comparable effects within a vocational education setting using fully immersive VR technology.

One key explanatory mechanism may lie in the embodied and interactive nature of VR environments. Unlike traditional screen-based instruction, immersive VR allows learners to manipulate virtual objects, navigate spatial representations, and receive immediate feedback within simulated systems. Such embodied interaction may strengthen mental model construction, facilitating deeper understanding of computational structures and logical relationships. This aligns with theoretical perspectives emphasizing active knowledge construction and situated cognition in technology-enhanced learning.

Additionally, the mission-based progression of tasks likely contributed to sustained cognitive engagement. By sequencing challenges from guided problem decomposition to more complex reasoning tasks, the intervention provided scaffolding that supported gradual development of CT skills. This structured complexity may explain why improvements were not limited to a single dimension but were observed across multiple components of computational thinking.

In a broader educational context, these findings suggest that VR game-based learning can function not merely as a motivational enhancement tool but as a cognitively meaningful instructional medium. Within vocational education, where applied reasoning and domain-specific problem-solving are central, immersive simulations appear particularly well suited to bridging abstract computational principles and real-world professional practices.

Taken together, the results contribute to the growing evidence that immersive learning technologies, when pedagogically aligned and cognitively scaffolded, can meaningfully support the development of computational thinking competencies in secondary vocational contexts.

### Dimension-specific insights into computational thinking development

Although all computational thinking (CT) dimensions improved significantly, the pattern of gains provides insight into how immersive VR environments may differentially support cognitive processes.

**Algorithmic Thinking** demonstrated the strongest improvement. The mission-based structure required students to construct, test, and refine logical sequences with immediate system feedback. Such iterative execution likely reinforced procedural reasoning and conditional logic, explaining the pronounced gains in this dimension. Similar effects have been observed in game-based environments that emphasize rule-based progression and structured challenges.

**Problem Decomposition** also showed substantial improvement, likely supported by the scaffolded design of the missions. By progressively increasing task complexity, the intervention encouraged students to break down problems into manageable components, strengthening analytical structuring skills.

In contrast, **Abstraction and Relevance Filtering**, although significantly improved, showed comparatively lower performance in the applied assessment. Abstraction requires identifying essential principles while filtering contextual detail. The rich and immersive nature of VR may increase cognitive load, potentially making abstraction more demanding without explicit instructional emphasis.

**Pattern Recognition** demonstrated balanced gains, suggesting that repeated exposure to structurally similar mission formats may have facilitated schema formation and identification of recurring logical patterns.

Overall, these findings suggest that immersive VR game-based learning may naturally reinforce procedural and sequential reasoning while still supporting, though to a slightly lesser extent, abstraction processes. This nuanced pattern contributes to understanding how VR-based interventions interact with distinct components of computational thinking.

### Pedagogical implications for vocational education contexts

The findings of this study have important implications for competency-based vocational education, particularly within Software Engineering and Game Development (PPLG) programs. Vocational curricula emphasize applied problem-solving, system design, and procedural accuracy—competencies closely aligned with computational thinking (CT). The observed improvements suggest that immersive VR game-based learning can serve as an effective medium for strengthening these core competencies in an authentic and practice-oriented manner.

First, the integration of structured, mission-based VR tasks demonstrates the value of aligning computational challenges with vocationally relevant scenarios. Embedding CT processes within software feature planning, workflow debugging, and system organization tasks appears to enhance perceived relevance and facilitate transfer between conceptual reasoning and domain-specific practice.

Second, the balanced gains across CT dimensions indicate that immersive environments can support holistic skill development when tasks are scaffolded progressively. For vocational settings, this suggests that VR should not merely replicate traditional instruction in 3D form but should be intentionally designed to sequence cognitive complexity and provide iterative feedback.

Third, the convergence between objective assessments and performance-based tasks highlights the importance of integrating applied evaluation methods within vocational education. Assessing students’ ability to operationalize CT in realistic problem contexts provides a more comprehensive representation of competency than selected-response testing alone.

Overall, the results suggest that VR game-based learning can function as a pedagogically aligned tool within vocational programs, supporting both conceptual understanding and authentic skill application when integrated with clear instructional scaffolding and domain relevance.

### Positioning within the broader literature on VR and game-based learning

The present findings are consistent with a substantial body of research demonstrating the positive educational impact of immersive VR. A meta-analysis by Wu reported that head-mounted immersive VR significantly enhances learning performance compared to less immersive formats. These effects are theoretically supported by the Cognitive Affective Model of Immersive Learning (CAMIL) developed by Makransky, ^31^ which explains how presence, emotional engagement, and active control can translate immersive experiences into measurable cognitive gains. The improvement in computational thinking (CT) observed in this study aligns with this mechanism, as learners interacted directly with simulated problems requiring structured reasoning.

More specifically, the results corroborate empirical work by, ^15^ who demonstrated that immersive VR mini-games can foster CT skills in higher education settings. Their design-based and experimental studies emphasize structured mini-game mechanics, iterative problem-solving, and scaffolded challenges—elements similarly embedded in the present intervention. Likewise, research by ^1^ shows that VR gaming environments can effectively support CT development when tasks are aligned with decomposition, abstraction, and algorithmic reasoning processes. The current study extends these findings by applying VR game-based CT learning within a vocational education context, where authentic and practice-oriented competencies are central.

Broader systematic reviews further contextualize these outcomes. Reviews by ^19^ ^,^ ^20^ highlight that VR’s effectiveness depends on pedagogical integration rather than technological novelty. Similarly, meta-analytic evidence in vocational and mixed-reality training ^17^ indicates positive cognitive and behavioural outcomes when immersive environments simulate authentic tasks. The present quasi-experimental findings support this claim, demonstrating that VR-based game mechanics can yield measurable CT improvements when aligned with competency-based vocational learning.

In sum, this study contributes to the literature by reinforcing theoretical models of immersive learning, empirically supporting VR game-based approaches for CT development, and extending prior higher-education-focused research into vocational education settings using performance-based assessment.

### Transferability and methodological value of the research design

Beyond its theoretical and empirical contributions, a key value of this study lies in the transferability of its underlying research and instructional design to other vocational sectors. Although the intervention was contextualized for Software Engineering and Game Development (PPLG), the core framework—which pairs Bebras-style computational logic with 3D immersive task simulations—can be readily adapted to diverse VET domains, such as industrial automation, smart manufacturing, automotive troubleshooting, or healthcare logistics. Adapting the approach requires substituting software-specific problems with domain-specific process models and industrial equipment interactions while preserving the scaffolded logic structure.

Furthermore, the research design itself serves as an empirical and methodological finding. Conducting rigorous technology-enhanced learning evaluations in VET often faces practical hurdles, including tight class schedules, shared lab infrastructure, and fixed curricula. This study demonstrates that a quasi-experimental, classroom-embedded VR research design can be successfully implemented using standalone headsets (e.g., Meta Quest 2) within intact classes and standard timetables without disrupting ongoing instruction. By balancing ecological validity with structured pre−/post-test and performance-based measures, this study offers a tested methodological blueprint for researchers and vocational educators seeking to evaluate immersive competency-based learning in authentic educational environments.

### Practical implications for instructional design and implementation

The findings suggest several practical implications for integrating VR game-based learning into vocational education. First, VR activities should be explicitly aligned with computational thinking (CT) constructs. As emphasized in prior VR mini-game research by Francisca J. Agbo, immersive environments are most effective when game mechanics directly require decomposition, abstraction, and algorithmic reasoning rather than focusing solely on engagement. Tasks should therefore embed structured problem-solving cycles and progressive challenges. ^15^ ^,^ ^33^

Second, authenticity is essential. Reviews by Radianti indicate that VR yields stronger outcomes when simulations mirror real-world professional contexts. ^19^ In vocational settings, integrating CT within industry-relevant scenarios enhances transferability to workplace competencies.

Third, cognitive load and scaffolding must be carefully managed. Based on the Cognitive Affective Model of Immersive Learning proposed by Makransky, immersive features should support—not distract from—core reasoning processes. Clear guidance and staged progression help maintain cognitive focus.

Finally, successful implementation requires teacher preparation and performance-based assessment strategies. VR systems can incorporate in-game analytics to evaluate students’ reasoning processes, aligning assessment with competency-based vocational education.

### Methodological considerations and study limitations

Several methodological considerations should be acknowledged when interpreting the findings. First, the study employed a quasi-experimental design, which limits the ability to establish full causal inference. Although pretest–posttest comparisons indicate significant improvements in computational thinking (CT), the absence of random assignment may introduce selection bias or uncontrolled contextual variables. Future randomized controlled designs would strengthen internal validity.

Second, the sample was drawn from a specific vocational education context, which may limit generalizability to other disciplines, age groups, or institutional settings. As noted in broader reviews of immersive VR in education, ^19^ contextual factors such as curriculum structure, technological infrastructure, and instructor readiness can influence outcomes.

Third, the duration of the intervention was relatively short. While measurable gains were observed, longer exposure may produce different patterns of development, particularly for higher-order CT dimensions such as abstraction. Longitudinal designs would allow examination of retention and transfer effects over time.

Fourth, although performance-based assessment was used, measurement of CT remains complex. Computational thinking involves both observable problem-solving behaviors and internal cognitive processes, which may not be fully captured through task-based scoring alone. Integrating process analytics or mixed-method approaches could provide richer insights.

Finally, technical factors such as device usability, familiarity with VR interfaces, and potential novelty effects may have influenced learner engagement. As highlighted in immersive learning models such as the one proposed by Makransky, engagement and presence can mediate learning outcomes, but these effects may vary as learners become accustomed to the technology. Taken together, these limitations suggest that while the findings are promising, they should be interpreted with caution and validated through broader, more controlled investigations.

### Directions for future research

Future research should strengthen causal evidence through randomized controlled trials, improving upon the quasi-experimental design used in this study. Longitudinal investigations are also needed to examine the retention and transfer of computational thinking (CT) skills over time. As suggested in immersive learning research by Makransky, ^31^ initial engagement effects may differ from long-term learning outcomes. Further studies should involve larger and more diverse vocational samples to enhance generalizability. Reviews by Radianti highlight that contextual and disciplinary factors can moderate VR learning effects.

Finally, future work should incorporate process-oriented analytics and comparative designs (e.g., immersive vs. non-immersive conditions) to better understand how and under what conditions VR game-based learning most effectively supports computational thinking development.

## Conclusion

This study investigated the impact of VR game-based learning on students’ computational thinking (CT) skills within a vocational education context using a quasi-experimental design and performance-based assessment. The findings demonstrate that immersive VR mini-games can significantly enhance overall CT performance, with improvements observed across key dimensions, including decomposition, abstraction, algorithmic thinking, and pattern recognition.

The results suggest that immersive VR environments support cognitive development when game mechanics are deliberately aligned with structured problem-solving processes. Rather than relying on technological novelty, the effectiveness of the intervention appears to stem from authentic task simulation, iterative challenges, and meaningful learner interaction within the virtual environment.

By situating VR-based computational thinking development within competency-based vocational learning, this study highlights the potential of immersive technologies to strengthen higher-order thinking skills in practice-oriented settings. Although methodological and contextual limitations should be considered, the findings provide empirical support for integrating well-designed VR game-based learning into vocational curricula to foster computational thinking competencies.

## Ethical considerations

This study was designed and conducted in accordance with ethical principles for educational research involving human participants. Ethical approval was granted by the Research Ethics Committee (Komisi Etik Penelitian), Directorate of Research and Community Service, Universitas Negeri Yogyakarta, Indonesia (Approval Number: T/64.20/UN34.9/PT.01.04/2025). Permission to conduct the study was also obtained from the participating vocational school, State Vocational High School 5 Surakarta.

Participation in the study was voluntary. Students were informed about the purpose of the study, the learning activities involved, and their right to withdraw at any time without academic penalty. As the participants were minors, written informed consent was obtained from their parents or legal guardians, in addition to the students’ assent.

All data were collected anonymously and treated confidentially. No personally identifiable information was included in the dataset or in any publications arising from the study. The VR learning activities were designed to align with regular instructional objectives and posed no known physical or psychological risks beyond those associated with standard classroom learning.

## Data Availability

All data underlying the results are available as part of the article and some supporting figures can be found in the figshare repository https://doi.org/10.6084/m9.figshare.32144917. ^34^ While the experiment resuts generated and analyzed during the current study also can be found in the fighsare repository https://doi.org/10.6084/m9.figshare.32144944. ^35^ Data are available under the terms of the Creative Commons Attribution 4.0 International license (CC-BY 4.0). This study was reported in accordance with the STROBE (Strengthening the Reporting of Observational Studies in Epidemiology) guidelines for observational research.
