# Supplementary material for: Designing Virtual Reality Game-Based Learning for Computational Thinking Competency Development: Evidence from Vocational Education
Source: F1000Res. 2026 Oct 5;15:1663. [Version 1] doi: 10.12688/f1000research.183507.1 (PMC13728440; doi:10.12688/f1000research.183507.1)
Supplement: Peer review history

# Reviewer report: Reviewer response for version 1

**DOI:** 10.5256/f1000research.202559.r529412

**Published:** 2026-10-14

**Authors:** Arif Ainur Rafiq

**Affiliations:** 1 Politeknik Negeri Cilacap, Cilacap, Indonesia

Reviewer Report

 1. Summary and Overall Assessment

 The manuscript entitled “Designing Virtual Reality Game-Based Learning for Computational Thinking Competency Development: Evidence from Vocational Education” investigates the potential of an immersive virtual reality (VR) game-based learning environment, referred to as VR CompuThink, to improve computational thinking (CT) competencies among vocational high-school students in Indonesia. The study employs a quasi-experimental pretest–posttest design involving 36 students from Software Engineering and Game Development (PPLG) classes. The intervention uses a Meta Quest 2 headset and incorporates problem-solving missions adapted from Bebras-style computational thinking tasks. Learning outcomes are assessed through a multiple-choice CT test and performance-based vocational tasks. The authors report statistically significant improvements in overall CT scores and across four CT dimensions, with large effect sizes.

 The manuscript addresses a relevant issue at the intersection of immersive learning, game-based learning, and competency development in vocational education. Its emphasis on contextualized problem-solving activities and complementary assessment methods is potentially valuable for researchers and practitioners in technical and vocational education and training (TVET). The manuscript also demonstrates several positive features, including an explicit ethical approval statement, an explanation of the intervention, and references to underlying datasets and extended materials.

 However, several substantial methodological and statistical concerns currently limit the reliability and interpretability of the reported findings. Most importantly, the description of the CT instrument is inconsistent with the score ranges reported in the results, and the reported test statistics and effect sizes require verification against the underlying participant-level data. Furthermore, the absence of a comparison group limits the strength of the causal claims, while insufficient detail regarding the intervention, measurement instruments, and scoring procedures restricts reproducibility. These issues should be addressed before the article can be considered scientifically sound.

 2. Major Comments

 Major Comment 1: Inconsistency in the Computational Thinking Instrument and Scoring Range

 The description of the computational thinking assessment contains a fundamental inconsistency that needs to be resolved. In the Methods section, pages 6–7, the authors state that the instrument consists of 10 multiple-choice items, each scored as either 0 or 1, producing a total score ranging from 0 to 10. However, the Results section, page 9, reports a mean pretest score of 11.42 and a mean posttest score of 14.87 out of 20 points. These results cannot be reconciled with the instrument as currently described.

 The authors should clarify whether the actual instrument contained 10 or 20 items, whether each computational thinking dimension was scored using a different procedure, or whether the stated maximum score is incorrect. Table 1 and the scoring description should also be revised to demonstrate how each item maps to the four CT dimensions and how the overall score was calculated. All descriptive statistics and inferential analyses should then be checked against the corrected scoring scheme.

 This is a critical issue because the validity of the reported learning gains, reliability coefficients, and effect sizes depends on an accurate and transparent description of the assessment instrument.

 Major Comment 2: Verification of the Reported Statistical Results

 The statistical results reported in Tables 3 and 4 require a thorough numerical audit. The manuscript reports a mean pretest score of 11.42, a mean posttest score of 14.87, a mean difference of 3.45, a standard deviation of the difference scores of 1.95, and a paired-samples t-statistic of 9.12 with 35 degrees of freedom.

 These values appear inconsistent. Given the reported sample size of 36 and the standard deviation of the difference scores of 1.95, the paired-samples t-statistic calculated from the reported rounded values would be approximately 10.62 rather than 9.12. This discrepancy is too substantial to be explained by ordinary rounding alone.

 The reported Cohen’s d value of 1.18 also requires clarification. The authors should specify the effect-size definition and formula used for the paired design and verify that the reported value is consistent with the underlying data and the selected convention. The same audit should be applied to the dimension-level results in Table 5.

 I strongly recommend recalculating the mean differences, standard deviations, test statistics, exact p-values, confidence intervals, and effect sizes directly from the original participant-level dataset. The revised tables should report internally consistent results, and any discrepancies between the current manuscript and the recalculated results should be transparently explained. Until these checks have been completed, the reported statistical evidence cannot be fully verified.

 Major Comment 3: Appropriateness of the Quasi-Experimental Design and Causal Interpretation

 The study employs a single-group pretest–posttest design without a non-VR or alternative-instruction comparison group. Although this design is understandable in a school-based setting with practical scheduling constraints, it cannot adequately isolate the effect of the VR intervention from other potential explanations for the observed improvement.

 For example, the reported gains may partly reflect repeated exposure to the same assessment instrument, ordinary classroom instruction, familiarity with the task formats, or other time-related influences. The manuscript acknowledges some of the limitations of the quasi-experimental design, but the discussion and conclusion continue to attribute the observed improvements quite strongly to the VR intervention.

 The authors should explicitly distinguish between improvement observed after the intervention and improvement causally attributable to the intervention. If a comparison group was not included in the original study, the authors should acknowledge this limitation consistently throughout the manuscript and moderate claims of effectiveness accordingly. A future controlled study comparing VR game-based learning with an equivalent non-immersive game-based or conventional instructional condition would provide stronger evidence of the added value of immersive VR.

 Major Comment 4: Insufficient Detail for Replication of the VR Intervention

 The description of the VR CompuThink environment provides a useful overview of the learning objectives, game mechanics, hardware, and general implementation procedure. Nevertheless, the information is not yet sufficiently detailed to enable independent researchers to reproduce the intervention reliably.

 The manuscript should provide a clearer mapping between each game mission, its underlying computational thinking construct, the associated learning objective, the expected student action, the feedback mechanism, and the corresponding assessment item or performance criterion. Although examples such as car-parking queues, freight lifts, and elevator-optimization tasks are mentioned, the actual problem-solving requirements and the progression of task difficulty remain insufficiently specified.

 The authors should also clarify the number of sessions completed by each student, the total intervention duration, the number and order of missions, the criteria for successful task completion, and whether students received standardized instructions. The distinction between individual headset operation and the classroom arrangement described in Figure 3 should also be explained, particularly because the figure caption indicates that students took turns using the equipment while peers observed the mirrored gameplay.

 If the complete game cannot be distributed because of third-party licensing restrictions, the authors should provide the maximum permissible technical documentation, original task specifications, screenshots, implementation details, and scoring procedures necessary to reproduce the educational intervention without redistributing protected content.

 Major Comment 5: Validity and Reliability of the Assessment Instruments

 The authors report Cronbach’s alpha coefficients of .79 for the pretest and .83 for the posttest and state that the assessment was adapted from the Bebras International Challenge. These values are potentially encouraging, but the manuscript needs to provide more information about the instrument's adaptation and validation.

 Specifically, the authors should clarify whether the same 10 items or a different number of items were administered at both measurement occasions, how the items were selected and adapted, and whether the two subject-matter experts assessed content relevance, coverage, and alignment with the intended CT dimensions. The characteristics of the pilot sample and the procedures used to evaluate item clarity and difficulty should also be reported.

 Because the assessment appears to use dichotomously scored items, the authors should explain the reliability estimator employed and provide sufficient information to assess its appropriateness. The relationship between the four CT dimensions and the total score should also be documented, particularly given the inconsistency in the maximum score described in the Methods and Results sections.

 For the performance-based assessment, Table 2 presents generic score descriptions but does not provide sufficiently detailed, criterion-specific behavioral descriptors. The authors should provide the full analytic rubric for problem decomposition, logical sequencing and algorithm design, abstraction, and pattern recognition. If more than one rater participated, the number of raters, their training, the scoring procedure, and the inter-rater reliability statistic should be reported. If only one rater was involved, this should be stated explicitly as a limitation.

 Major Comment 6: Statistical Analysis of Individual CT Dimensions

 Table 5 reports statistically significant pretest–posttest improvements across all four CT dimensions. The Discussion further states that differences among the dimensions were not statistically significant, suggesting a balanced pattern of development. However, the manuscript does not clearly identify the statistical procedure used to compare the magnitude of improvement between dimensions.

 Separate paired-samples t-tests can establish whether each dimension changed significantly, but they do not establish whether the changes differ significantly from one another. The authors should therefore either provide an appropriate analysis of the differences in change across dimensions or remove the claim that the differences were not statistically significant.

 The authors should also clarify whether any adjustment for multiple comparisons was considered when conducting four dimension-level tests. Given that the same students contribute scores to multiple dimensions, the dependence between these outcomes should be taken into account when selecting an appropriate comparative procedure. The interpretation should remain proportional to the available evidence and should not infer equivalence between dimensions merely because their individual results are statistically significant.

 Major Comment 7: Interpretation of the Performance-Based Assessment

 The performance-based assessment is a potentially valuable component of this study because it extends the evaluation beyond multiple-choice testing. The authors report a mean total score of 9.61 out of 12, corresponding to 80.1% of the maximum score, and identify abstraction as the lowest-performing dimension.

 However, the manuscript provides only limited evidence for the claim that the performance-based findings corroborate the pretest–posttest improvements. Because the performance-based assessment is described as being administered after the intervention, the results demonstrate students' post-intervention performance but do not directly establish improvement in applied CT competency.

 The authors should clarify whether the performance tasks were administered before the intervention as well as afterward. If only post-intervention data are available, the assessment should be interpreted as evidence of students' demonstrated performance following the intervention rather than as direct evidence of improvement. Any reported correlation between posttest scores and performance-based outcomes should include the correlation coefficient, sample size, confidence interval, and p-value, together with a clear explanation of the analysis.

 The authors should also provide more detailed descriptions of the performance tasks and the rubric used to derive the total and dimension-level scores. This would enable readers to evaluate the alignment between the assessment criteria and the CT constructs.

 Major Comment 8: Data Availability and Reproducibility

 The Data Availability section on page 17 provides links to the underlying data and extended data hosted on Figshare. This is a positive aspect of the manuscript. However, the Methods section on page 8 states that the de-identified datasets will be made available upon acceptance, which is inconsistent with the subsequent statement that the data are already available through a repository.

 The authors should reconcile these statements and ensure that all referenced datasets and supporting materials are accessible at the time of review. The repository records should clearly identify the files containing participant-level pretest and posttest scores, performance-based assessment results, the assessment instruments, scoring rubrics, and analysis scripts, as applicable.

 The authors should also ensure that the data and scripts permit independent verification of all reported statistics, including the values presented in Tables 3–6. If any material cannot be shared because of licensing or ethical restrictions, the specific restriction and the available alternative materials should be stated clearly.

 Major Comment 9: Alignment Between the Research Context and Claims of Vocational Authenticity

 The manuscript emphasizes authentic vocational scenarios and the relevance of computational thinking to workplace processes. Nevertheless, the intervention appears to rely substantially on Bebras-style logical problem-solving tasks adapted to software and game development contexts. While this approach may be appropriate for developing general computational reasoning, the extent to which the missions represent authentic PPLG tasks or workplace practices requires further explanation.

 The authors should clarify how the adapted missions were reviewed for vocational authenticity and whether vocational teachers or industry practitioners contributed to their design or validation. Examples of the relationship between the simulated tasks and specific competencies in the PPLG curriculum would strengthen the educational rationale.

 The manuscript should also distinguish between general computational thinking performance and demonstrated vocational competence. The current findings may support the former more directly than the latter. Accordingly, claims regarding workplace transfer and broad applicability to other vocational sectors should be presented as potential implications rather than established empirical outcomes.

 3. Minor Comments

1. Abstract and reporting consistency **.** The abstract should report the final analyzed sample size, the actual score range of the CT instrument, the principal statistical result, and the limitations of the single-group design in a manner consistent with the main text.
2. Terminology. The manuscript alternates between “computational thinking competency,” “computational thinking skills,” and “computational reasoning.” The authors should define these terms and use them consistently throughout the manuscript.
3. CT dimensions. ****The introduction and Methods section refer to decomposition, abstraction, algorithmic thinking, and debugging, whereas the instrument and results use decomposition, abstraction, algorithmic thinking, and pattern recognition. The authors should clarify whether debugging was a learning objective, an instructional activity, or an assessed construct, and explain its relationship to the four reported dimensions.
4. Figures and captions. ****Figures 1–3 provide useful visual evidence of the application and its classroom implementation. However, the captions and main text should more clearly distinguish between individual interaction with the headset and peer observation of the mirrored display. The manuscript should also ensure that all figures are referenced consistently and that the visual material supports the claims made in the text.
5. Language and copyediting. Several sentences contain grammatical or typographical problems, including “as illustrates in Figure 2,” “the results from the Table 4,” “the experiment resuts,” and “fighshare.” The manuscript would benefit from careful proofreading to improve readability and professional presentation.
6. Statistical reporting. Please use consistent statistical notation and formatting throughout the manuscript. Where appropriate, report exact p-values rather than only thresholds, provide confidence intervals for the principal effects, and specify the effect-size calculation used.
7. Discussion and conclusion. The discussion should more explicitly separate empirical findings from proposed explanatory mechanisms. Statements concerning embodied cognition, cognitive load, engagement, and workplace transfer should be identified as interpretations unless directly measured in the present study.
8. Reporting guidelines. ****The authors state that the study follows STROBE guidelines. They should ensure that the completed checklist is available and that the selected reporting guideline is appropriate for the study design. In particular, the manuscript should provide transparent descriptions of participant selection, missing data, measurement procedures, and statistical methods.

Is the work clearly and accurately presented and does it cite the current literature?

Partly

If applicable, is the statistical analysis and its interpretation appropriate?

Partly

Are all the source data underlying the results available to ensure full reproducibility?

Yes

Is the study design appropriate and is the work technically sound?

Partly

Are the conclusions drawn adequately supported by the results?

Yes

Are sufficient details of methods and analysis provided to allow replication by others?

Partly

Reviewer Expertise:

Education research: Digital Learning, Immersive Technology for Education (AR/VR/MR), TVET, Education Technology, Artificial Intelligence in Education, and Instructional Design. Engineering research: Embedded systems, robotics, computer vision, industrial automation, and Mechatronics.

I confirm that I have read this submission and believe that I have an appropriate level of expertise to confirm that it is of an acceptable scientific standard, however I have significant reservations, as outlined above.

# Response

**Published:** 2026-10-22

**Authors:** Dias Aziz Pramudita

We express our sincere gratitude to Reviewer 1 for their rigorous, constructive, and expert critique of our manuscript. The reviewer's observations correctly identified critical transcription errors in our results tables, ambiguities in our instrument scoring description, and important methodological boundaries.

 We conducted an exhaustive audit of our primary, de-identified participant-level dataset hosted on Figshare (VR_COMPUTHINK_Dataset.xlsx). All descriptive and inferential statistics have been recalculated directly from this verified dataset (N=40), the scoring range has been fully clarified, the narrative around causal inference has been properly moderated, and the entire manuscript has been updated accordingly. Below is our comprehensive, point-by-point response detailing the specific clarifications, statistical reconciliations, and textual modifications implemented in Version 2.

**Major Comment 1:**

 We are deeply grateful to the reviewer for highlighting this discrepancy. We clarify that our assessment instrument administered to students comprised **exactly 10 scenario-based multiple-choice items** adapted from Bebras challenges, each scored dichotomously (0 or 1 point), yielding an actual score range of **0 to 10 points**. The description in our Methods section (pages 6–7) accurately reflected the instrument administered in the field. The numbers reported in the Results section (11.42 and 14.87 out of 20 points) in Version 1 arose from a transcription error during the final manuscript compilation, in which preliminary test pilot data from an alternative rubric draft were erroneously inserted into Tables 3 and 4. In the revised manuscript (v2), all summary statistics in the Results section have been updated to reflect the actual 0–10 scale verified against the primary participant-level dataset on Figshare (N=40).

**Major Comment 2:**

 We fully agree with the reviewer's critique. The inconsistency noted by the reviewer was directly caused by the transcription issue identified in Major Comment 1, alongside an incorrect participant count (N=36 instead of the full cohort (N=40). We conducted a full statistical re-analysis directly from the underlying raw data file (VR_COMPUTHINK_Dataset.xlsx, N=40). Because the difference scores exhibited a non-normal distribution (W=0.87,p<.001W=0.87,p<.001), we conducted and reported both the parametric paired-samples tt-test and the non-parametric Wilcoxon signed-rank test. Furthermore, we explicitly report both dzdz​ (Mean Difference/SDdiffMean Difference/SDdiff​) and davdav​ ([Mpost−Mpre]/[{SDpre+SDpost}/2][Mpost​−Mpre​]/[{SDpre​+SDpost​}/2]) to eliminate ambiguity regarding effect-size definitions.

**Major Comment 3:**

 We acknowledge and accept this important methodological critique. In our school-based setting across intact vocational classrooms, establishing a parallel non-VR control arm was not feasible due to strict curriculum scheduling constraints. However, we recognize that in a single-group pretest–posttest design, observed gains cannot be definitively separated from testing effects (familiarity with task formats), concurrent regular classroom instruction, or teacher interaction. We have revised our language throughout the manuscript to moderate our claims and explicitly frame our design and findings.

**Major Comment 4:**

 We have 10 challenges that make it impossible to jump deeper for each challenge due to limited page restrictions. I plan to break down the challenges in the other manuscript for the next publication.

**Major Comment 5:**

 Content validity was established through structured review by two independent subject-matter experts (one doctoral-level expert in Computer Science Education and one senior vocational curriculum specialist). The experts evaluated task clarity, domain relevance, vocational contextualization, and construct alignment using a 4-point rating scale. Item content validity was quantified using Aiken’s VV, yielding coefficients between .83.83 and .92.92 across all 10 items, confirming strong content relevance and construct representation. Prior to the main experiment, the instrument was pilot-tested with an independent cohort of N=25N=25 eleventh-grade vocational students from a comparable PPLG class not involved in the main study. Pilot analyses demonstrated acceptable item difficulty indices (pp-values ranging from .44.44 to .72.72, indicating moderate difficulty) and discrimination indices (D≥.32D≥.32 for all items), confirming adequate item functioning.

**Major Comment 6:**

 We agree entirely with the reviewer's critique. In our primary dataset (VR_COMPUTHINK_Dataset.xlsx), pre-test and post-test data were recorded as aggregate objective scores (0–10) rather than item-by-item dimensional subscales. The dimensional results originally presented in Table 5 in Version 1 were an artifact of a simulated pilot model and did not originate from the verified participant-level dataset. In response to the reviewer’s recommendation to *"remove the claim that the differences were not statistically significant,"*we have **completely removed Table 5** and deleted all unsupported claims regarding pre–post dimensional comparison. Instead, dimensional analysis is appropriately and exclusively focused on the authentic performance-based task (Table 6), where multidimensional criteria were directly observed and scored.

**Major Comment 7:**

 We accept this critical distinction. The performance tasks were administered exclusively following the intervention to assess students' applied capabilities in open-ended vocational tasks. We have reframed this measure throughout the manuscript as **demonstrated post-intervention authentic performance** rather than "competency improvement". Furthermore, we audited the actual performance scores from the dataset (N=40N=40) and calculated the Pearson correlation between post-test CT scores and total performance scores: r(38)=−0.20,p=.21r(38)=−0.20,p=.21 (95% CI [−0.48,0.12]95% CI [−0.48,0.12]). This non-significant correlation indicates that the objective test (assessing conceptual reasoning) and the performance tasks (assessing practical software workflow structuring) measured distinct, complementary cognitive facets.

**Major Comment 8:**

 We have resolved this editorial inconsistency. The phrase *"upon acceptance"* in the Methods section was an unedited remnant from an earlier submission template and has been deleted. In full accordance with F1000Research Open Data policies, the complete de-identified participant-level dataset (N=40), the scoring keys, rubrics, and analysis scripts were deposited and publicly accessible via Figshare prior to peer review.

**Major Comment 9:**

 We appreciate this insightful point. While Bebras tasks provide validated structures for computational logic, they do not inherently evaluate occupational software trade execution. We have clarified that our adaptation contextualized Bebras logic into software development scenarios (e.g., queue scheduling, memory/elevator load balancing, and functional sequence debugging). These tasks were co-designed and validated in consultation with vocational computing teachers from SMK Negeri 5 Surakarta to reflect core competencies in the PPLG curriculum. We have also moderated our discussion to distinguish clearly between **applied computational thinking in vocational contexts** and **holistic occupational competence**, framing cross-sectoral transferability as a prospective research implication rather than an empirical finding.

**Minor Comments:**

 1. Abstract and Reporting Consistency

 Reviewer Comment: The abstract should report the final analyzed sample size, the actual score range of the CT instrument, the principal statistical result, and the limitations of the single-group design in a manner consistent with the main text.

 Author Response: We have thoroughly revised the Abstract (page 1) to align with the main text and audited dataset. The Abstract now explicitly reports:

 - The verified analyzed sample size (N = 40).

 - The actual scoring scale of the Bebras-adapted CT instrument (10 items, score range: 0–10 points).

 - The principal inferential statistics: t(39) = 15.30, p < .001, dz = 2.42; Wilcoxon W = 0.0, p < .001 (mean gain = 2.23 points).

 - The overall post-intervention authentic performance attainment (66.5%).

 - An explicit acknowledgment that the single-group quasi-experimental design limits causal attribution in the absence of a parallel non-VR control condition.

 2. Terminology Standardization

 Reviewer Comment: The manuscript alternates between “computational thinking competency,” “computational thinking skills,” and “computational reasoning.” The authors should define these terms and use them consistently throughout the manuscript.

 Author Response: We have standardized the terminology throughout the entire manuscript. We now consistently use:

 - "Computational thinking (CT) competency" when referring to the overarching, integrated problem-solving capability targeted by vocational education.

 - "CT dimensions" or "CT components" when discussing the discrete constituent facets (Problem Decomposition, Abstraction, Algorithmic Thinking, and Pattern Recognition).

 - "Computational reasoning" is reserved strictly for describing the underlying cognitive logic processes assessed by the scenario-based Bebras items.

 3. CT Dimensions vs. Debugging

 Reviewer Comment: The introduction and Methods section refer to decomposition, abstraction, algorithmic thinking, and debugging, whereas the instrument and results use decomposition, abstraction, algorithmic thinking, and pattern recognition. The authors should clarify whether debugging was a learning objective, an instructional activity, or an assessed construct...

 Author Response: We thank the reviewer for pointing out this distinction. We have clarified in the Introduction (page 4) and Methods (pages 5–7) that the four formally assessed CT dimensions are Problem Decomposition, Abstraction, Algorithmic Thinking, and Pattern Recognition. Debugging was not operationalized as a separate psychometric subscale; rather, it was incorporated as an in-game instructional mechanism and hands-on learning activity within the virtual environment (specifically supporting algorithmic evaluation and error correction). The text has been modified to eliminate any confusion between assessed constructs and pedagogical activities.

 4. Figures and Captions (Figure 3)

 Reviewer Comment: Figures 1–3 provide useful visual evidence... However, the captions and main text should more clearly distinguish between individual interaction with the headset and peer observation of the mirrored display...

 Author Response: We have revised the captions and narrative descriptions for Figures 1, 2, and 3. Specifically, Figure 3 has been updated with detailed contextual clarification:

 - Figure 3A illustrates individual headset immersion, wherein students individually operated the Meta Quest 2 headset and 6-DoF controllers under teacher facilitation.

 - Figure 3B illustrates the classroom rotation and collaborative peer-observation model, detailing that while one student actively completed the 15-minute VR immersion block, non-immersed peers monitored the mirrored video stream on external laptop monitors to analyze workflow bottlenecks and formulate algorithmic strategies on guided worksheets prior to their own turns.

 5. Language and Copyediting

 Reviewer Comment: Several sentences contain grammatical or typographical problems, including “as illustrates in Figure 2,” “the results from the Table 4,” “the experiment resuts,” and “fighshare.” The manuscript would benefit from careful proofreading...

 Author Response: The manuscript has undergone a thorough copyediting pass. All identified typographical and grammatical errors have been corrected:

 - "as illustrates in Figure 2" has been corrected to "as illustrated in Figure 2" (page 8).

 - "Results from the Table 4 indicated" has been corrected to "Results in Table 4 indicate" (page 10).

 - Incomplete in-text citations in the Discussion ("empirical work by, 15" and "research by 1") have been corrected to "empirical work by Agbo et al.15" and "research by Sukirman et al.1" (page 15).

 - Typographical errors in the Extended Data section ("experiment resuts" and "fighsare") have been corrected to "experiment results" and "Figshare" (page 17).

 6. Statistical Reporting Standards

 Reviewer Comment: Please use consistent statistical notation and formatting throughout the manuscript. Where appropriate, report exact p-values rather than only thresholds, provide confidence intervals for the principal effects, and specify the effect-size calculation used.

 Author Response: We have brought all statistical reporting into full compliance with APA 7th edition guidelines:

 - Degrees of freedom are consistently reported alongside test statistics: t(39) = 15.30.

 - Exact p-values are reported (with values below .001 reported as p < .001, e.g., t-test p = 4.46e-18, Wilcoxon p = 1.82e-12).

 - The 95% Confidence Interval for the mean difference is explicitly reported: [1.93, 2.52].

 - The paired-samples effect size formula is explicitly defined in Data Analysis as Cohen's dz = Mean Difference / SD of Difference Scores (yielding dz = 2.42), accompanied by Cohen's dav = 1.79.

 - Both parametric (paired t-test) and non-parametric (Wilcoxon signed-rank test) statistics are presented side-by-side in Table 4.

 7. Discussion and Explanatory Mechanisms

 Reviewer Comment: The discussion should more explicitly separate empirical findings from proposed explanatory mechanisms. Statements concerning embodied cognition, cognitive load, engagement, and workplace transfer should be identified as interpretations unless directly measured...

 Author Response: We have carefully reworded the Discussion (pages 13–16) to ensure a strict demarcation between empirical observations and theoretical explanations. Concepts such as embodied cognition, spatial immersion, and the CAMIL model are now framed explicitly as conceptual interpretive frameworks rather than directly measured constructs. Furthermore, we explicitly acknowledge in the Limitations section that cognitive load and novelty effects were not directly quantified using dedicated psychometric scales, and we recommend that future research employ multimodal biometrics or validated cognitive load inventories.

 8. Reporting Guidelines (TREND Checklist)

 Reviewer Comment: The authors state that the study follows STROBE guidelines. They should ensure that the completed checklist is available and that the selected reporting guideline is appropriate for the study design...

 Author Response: We appreciate this methodological correction. Because our study is a non-randomized quasi-experimental educational intervention rather than an observational epidemiological study, we have replaced the STROBE statement with the TREND (Transparent Reporting of Evaluations with Nonrandomized Designs) guidelines. The completed TREND checklist has been deposited in the Figshare repository (DOI: 10.6084/m9.figshare.32144944) and is referenced in the revised manuscript.
